# Complete Genome Sequence of *Capripoxvirus* Strain KSGP 0240 from a Commercial Live Attenuated Vaccine

**DOI:** 10.1128/genomeA.01114-16

**Published:** 2016-10-20

**Authors:** Frank Vandenbussche, Elisabeth Mathijs, Andy Haegeman, Ahmad Al-Majali, Steven Van Borm, Kris De Clercq

**Affiliations:** a Molecular Platform, Veterinary and Agrochemical Research Centre, Ukkel, Belgium; b Viral Diseases, Vesicular and Exotic Diseases, Veterinary and Agrochemical Research Centre, Ukkel, Belgium; c Jordan Bio-Industries Centre (JOVAC), Amman, Jordan; d Faculty of Veterinary Medicine, Jordan University of Science and Technology, Irbid, Jordan

## Abstract

Capripoxviruses cause economically important diseases in domestic ruminants in regions endemic for these viruses. We report here the complete genome sequence of the KSGP 0240 vaccine strain from the live attenuated vaccine Kenyavac (JOVAC).

## GENOME ANNOUNCEMENT

Viruses belonging to the *Capripoxvirus* genus, namely, goatpox virus (GTPV), sheeppox virus (SPPV), and lumpy skin disease virus (LSDV), cause important diseases in goats, sheep, and cattle, respectively. Vaccination plays a crucial role in limiting the diseases in regions that are endemic for the viruses. In these regions, the Kenyan SPPV and GTPV KSGP 0240 strain are widely used for vaccination against GTPV and SPPV. Although the virus was isolated from sheep, phylogenetic analyses of partial sequences have identified KSGP 0240 as a lumpy skin disease virus (1, 2). Here, we determined the complete sequence of the KSGP 0240 strain from the Kenyavac vaccine (JOVAC) directly from a commercial vaccine batch.

DNA was purified from a freeze-dried vaccine pellet dissolved in 3 ml of phosphate-buffered saline (PBS) using the Puregene core kit A (Qiagen), according to the manufacturer’s instructions. Presequencing enrichment was performed through an in-house long-range PCR methodology covering the entire genome with overlapping ~5.5-kb amplicons. P6-C4 sequencing was performed on a single-molecule real-time (SMRT) cell on a PacBio RSII sequencer (Pacific Biosciences) at the Genomics Core UZ Leuven (Leuven, Belgium).

Consensus amplicon sequences were obtained from the reads using the LAA-protocol (default parameters; Pacific Biosciences) in SMRT Portal (Pacific Biosciences) version 2.3.0. These amplicons were further assembled into a unique contig using the iAssembler software (3). Discrepancies with previously published LSDV genomes were confirmed by Sanger sequencing. The protein-coding genes were predicted by NCBI ORF-Finder (http://www.ncbi.nlm.nih.gov/orffinder/) and by GATU relative to the Neethling NI-2490 reference strain (accession no. NC_003027) (4).

Consensus amplicon sequences assembled into a single double-stranded linear DNA sequence of 150,663 bp, with an average G+C content of 25.90%. The strain KSGP 0240 included in the Kenyavac vaccine contains a 145,938-bp central coding region flanked by two identical terminal inverted repeats of at least 2,192 bp. The KSGP 0240 genome shares 99.9% homology with the LSDV strain Neethling NI-2490, differing by only a single amino acid substitution (P/Q in LSDV049) and a single-nucleotide deletion resulting in a frameshift that truncates the LSDV134 putative gene. Based on the complete genome sequence, the strain KSGP 0240 was confirmed to be an LSDV.

### Accession number(s).

The complete genome sequence of LSDV strain KSGP 0240 (Kenyavac vaccine) has been deposited in GenBank under accession number KX683219.
